# Spin‐Crossover in a Dinuclear Iron(II) Complex on Highly Oriented Pyrolytic Graphite: An X‐Ray Absorption Spectroscopy Study

**DOI:** 10.1002/cphc.202401081

**Published:** 2025-05-07

**Authors:** Marcel Walter, Sebastien Elie Hadjadj, Clara Trommer, Jorge Torres, Jendrik Gördes, David Swerev, Tauqir Shinwari, Christian Lotze, Chen Luo, Florin Radu, Felix Tuczek, Sangeeta Thakur, Wolfgang Kuch

**Affiliations:** ^1^ Institut für Experimentalphysik Freie Universität Berlin Arnimallee 14 14195 Berlin Germany; ^2^ Present address: Materials Physics Center CSIC ‐ UPV/EHU Manuel de Lardizabal 5 20018 San Sebastián Spain; ^3^ Institut für Anorganische Chemie Christian‐Albrechts Universität zu Kiel 24098 Kiel Germany; ^4^ Present address: Paul‐Drude‐Institut für Festkörperelektronik Institut im ForschungsverbundBerlin e.V. Hausvogteiplatz 5‐7 10117 Berlin Germany; ^5^ Helmholtz Zentrum Berlin für Materialien und Energie Albert‐Einstein Straße 15 12489 Berlin Germany

**Keywords:** dinuclear spin‐crossover complexes, light‐induced excited spin‐state trapping, pulsed layer injection, soft‐X‐ray‐induced excited spin‐state trapping, X‐ray absorption spectroscopy

## Abstract

The spin‐crossover (SCO) properties of the dinuclear complex $\left[{\left\{\text{Fe}{\left(H_{2}B{\left(\text{pz}\right)}_{2}\right)}_{2}\right\}}_{2}\mu -({\text{ac(bipy)}}_{2})\right]$ are studied as (sub)‐monolayer and thin film deposited by an ultrahigh vacuum liquid‐jet deposition technique on highly oriented pyrolytic graphite (HOPG) by X‐ray absorption spectroscopy. A comparison of the SCO properties of thin films and a dropcast sample indicates that the spin‐switching probability of the thin films is limited due to substrate–molecule interactions. The maximum percentage of molecules in the low‐spin (LS) state observed for 0.7 and 1.8 monolayers (ML) is ≈43% at a temperature of 80 K in comparison to the dropcast sample where ≈66% of the complex is in the LS state. The similar switching properties of the dropcast sample as of a bulk powder sample confirm that the SCO properties are not affected by the presence of solvent necessary for deposition. The soft‐X‐ray‐induced excited spin‐state trapping (SOXIESST) effect is pronounced in all samples, although the light‐induced high‐spin (HS) fraction of the dropcast and the thin‐film samples on HOPG is higher as compared to the HS fraction attained by SOXIESST, which confirms the sensitivity of the complex to light.

## Graphical abstract

This study investigates a spin‐crossover dinuclear Fe(II) complex deposited as a thin film under ultrahigh‐vacuum conditions by a pulsed layer injection technique. Its temperature‐dependent switching behavior, light‐induced excited spin‐state trapping, soft‐X‐ray‐induced excited spin‐state trapping, and temperature‐ and light‐induced spin crossover transitions are examined using X‐ray absorption spectroscopy. These characteristics are compared to the bulk material.

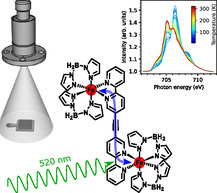

## 1 Introduction

The spin‐crossover (SCO) effect was discovered in the early 30 s of the 20th century by Cambi and Szegö.^[^ ^1^ ^]^ The first discovery of an Fe(II) complex undergoing a temperature‐induced SCO, switching between a low‐spin (LS) and a high‐spin (HS) state, was made in the mid‐1960s by Baker and Bobonich.^[^ ^2^ ^]^ The effects of temperature‐, light‐, and pressure‐induced switching have been investigated since then.^[^ ^3^, ^4^, ^5^, ^6^ ^]^ They make SCO materials attractive for use in memory devices,^[^ ^7^ ^]^ displays,^[^ ^8^, ^9^, ^10^ ^]^ sensors,^[^ ^11^ ^]^ molecular electronics,^[^ ^12^ ^]^ conductors,^[^ ^13^ ^]^ and terahertz technology.^[^ ^14^ ^]^ SCO properties of complexes adsorbed on surfaces make them interesting for device applications like data storage.^[^ ^15^, ^16^, ^17^ ^]^ However, the SCO properties can be affected by the choice of the substrate and by the method of deposition.^[^ ^18^, ^19^ ^]^ There are various reports on thin‐film deposition using the thermal evaporation method, demonstrating the preservation of SCO properties on surfaces with low density of states near the Fermi level, like highly oriented pyrolytic graphite (HOPG).^[^ ^5^, ^20^, ^21^, ^22^, ^23^ ^]^ The SCO properties are usually disturbed when the complex is deposited on metallic surfaces like Au(111) due to a strong interaction of the substrate with the complexes.^[^ ^24^ ^]^ Because of the preservation of SCO properties on HOPG, numerous SCO complexes have been explored on HOPG in detail, from submonolayers to thick layers.^[^ ^5^, ^20^, ^21^, ^22^, ^23^ ^]^

The next challenging step in the field of SCO complexes is to deposit molecules with two or more metallic centers on surfaces. Multicenter SCO complexes have been studied in bulk form.^[^ ^25^, ^26^ ^]^ An important finding from the first report on the dinuclear complex $\left[{\left\{\text{Fe(bt)}{\left(\text{NCS}\right)}_{2}\right\}}_{2}(\text{bpym})\right]$ is that the thermal SCO exhibits two steps, corresponding to SCO between LS–LS, LS–HS, and HS–HS,^[^ ^27^ ^]^ which was also supported recently by DFT calculations.^[^ ^28^ ^]^ Moussa et al. studied the dinuclear complex $\left[{\left\{\text{Fe(bt)}{\left(\text{NCS}\right)}_{2}\right\}}_{2}(\text{bpym})\right]$ by Raman spectroscopy and concluded that the SCO from LS to HS passes through a plateau, which is the result of the metal centers in the dinuclear complex being in different states (HS–LS).^[^ ^29^ ^]^ Depositing dinuclear complexes on surfaces, however, is challenging as the complexes are rather large and, therefore, they are not suitable for thermal evaporation. To deposit those molecules, one can apply a solvent method, where molecules dispersed in a solution are spray injected onto the surfaces in ultrahigh vacuum (UHV). This bears the possibility of coadsorption of solvent molecules along with the SCO molecules, which could affect the surface coverage and the SCO properties by an interaction of the solvent with the complex.^[^ ^30^, ^31^ ^]^

**Figure 1:**
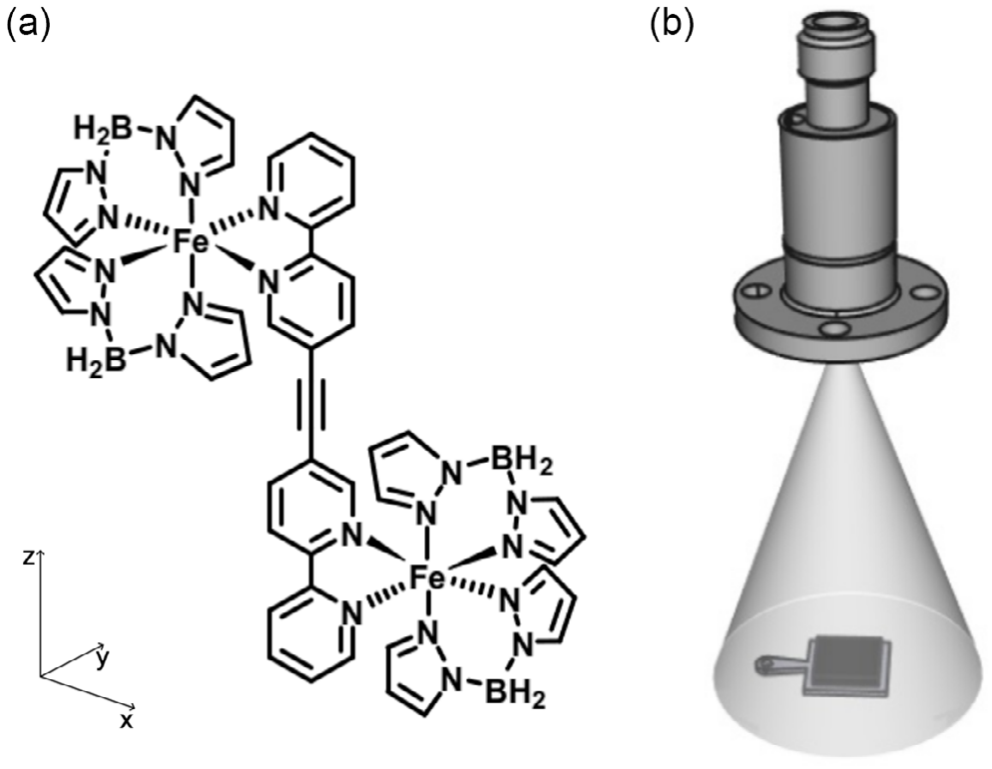
a) Structural formula of $\left[{\left\{\text{Fe}{(H_{2}B{\left(\text{pz}\right)}_{2})}_{2}\right\}}_{2}\mu -({\text{ac(bipy)}}_{2})\right]$. b) Simplified representation of the PLI setup. The pulse valve is shown on top with a cone of injected solvent, and the sample plate with the substrate is placed under the valve.

Here, we use SCO molecules in solution for the spray deposition onto a solid surface. To explore the SCO properties of a bigger complex, we are going to focus on linking two Fe centers within one, discrete, molecule (**Figure** **1**). The $[{\left\{\text{Fe}(H_{2}B{{\left(\text{pz}\right)}_{2})}_{2}\right\}}_{2}\mu -(\text{ac}{\left(\text{bipy}\right)}_{2})]\;$(Fe‐bipyacbipy) complex possibly decomposes at a temperature necessary for thermal evaporation. Therefore, and due to its molecular weight, an evaporation technique at room temperature is used to deposit the molecules that are dispersed in a solvent under UHV conditions. The Fe‐bipyacbipy complex has been studied in bulk by temperature‐dependent “physical property measurement system” (PPMS) and X‐ray absorption spectroscopy (XAS) and exhibits an LS‐to‐HS SCO as a function of temperature and light exposure.^[^ ^25^ ^]^ PPMS measurements on the Fe‐bipyacbipy complex show a steep SCO with temperature, indicating that all molecules make a direct SCO from the LS–LS to the HS–HS state,^[^ ^25^ ^]^ although XAS measurements on a bulk sample show that some fraction of the complex remains in the LS state.^[^ ^25^ ^]^ XAS measurements also revealed that the complex is switching to the HS state at low temperature due to soft‐X‐ray‐induced excited spin‐state trapping (SOXIESST) during X‐ray exposure.

In this report, we study thin films of the Fe‐bipyacbipy complex deposited on HOPG using a pulsed layer injection (PLI) system. The effect of temperature, light, and SOXIESST are studied in detail on thin films and a dropcast sample, also prepared on HOPG. The light‐induced excited spin‐state trapping (LIESST)^[^ ^32^ ^]^ is investigated at 10 K and the relaxation of the photo‐excited HS state to the LS state is activated by temperature, where *T*(LIESST) is the temperature above which the LS‐to‐HS state photoexcitation cannot be observed anymore.^[^ ^33^ ^]^ At *T*(LIESST) both, the temperature‐independent region and the thermally activated region are intrinsically combined.^[^ ^34^ ^]^ The various factors that affect the lifetime of the metastable HS state are well captured by *T*(LIESST) analysis.^[^ ^34^ ^]^

## 2 Results and Discussion

### 2.1 Temperature Dependence

**Figure 2:**
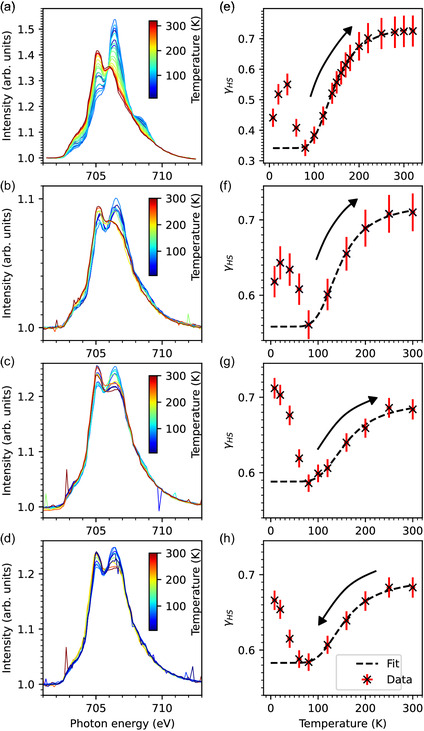
L_3_‐edge Fe^2+^ spectra for temperatures from 8 to 320 K of a) dropcast sample (3 ML), b) 0.7 ML and c) 1.8 ML heating, and d) 1.8 ML cooling cycle 300 to 8 K. Spectra (a)–(c) show a temperature‐induced LS‐to‐HS and (d) a HS‐to‐LS SCO. e–h) The corresponding HS fractions as a function of temperature. The fits (dashed lines) are based on the modified van't Hoff Equation (1) from the relaxation temperature to room temperature. The fit parameters are shown in Table 1. This data has been measured at the VEKMAG endstation.

The XAS spectra in **Figure** **2**a–d show the Fe *L*_3_ edge of Fe‐bipyacbipy at temperatures from 8 to 300 K at thermal equilibrium for the dropcast sample and two thin‐film samples. The thickness of the samples is estimated by comparing to the Fe *L*_3_ peak intensity of the mononuclear parent complex on HOPG.^[^ ^22^ ^]^ Using this method, the coverage for the different samples is determined as 3 ML for the dropcast, 0.7 ML for the thin‐film sample 1, and 1.8 ML for the thin‐film sample 2. If the molecules are oriented vertically rather than flat on the surface, the ratio between peak intensity and coverage may differ in the case of Fe‐bipyacbipy. Topographic atomic force microscopy (AFM) measurements have been performed to identify the orientation of the molecules. Figure S1, Supporting Information shows the surface scan of a 1.8 ML sample with a nanoisland on a relatively uniform surface and height jumps in the range of 1 nm, which is close to the average height of the mononuclear complexes ${\text{Fe(bpz)}}_{2}(\text{phen)}$ (0.7 nm) and ${\text{Fe(bpz)}}_{2}(\text{bipy})$ (bpz = hydrobis(pyrazolyl)borate) (1 nm), respectively.^[^ ^20^, ^22^ ^]^ This confirms a flat deposition of the molecules. Figure S2, Supporting Information shows AFM images of the dropcast sample, which indicate the formation of islands with an average height of about 10 nm.

The HS and LS spectra show maximum intensity at 705.2 and 706.5 eV, respectively. In Figure 2e–g, the corresponding high‐spin fractions ($\gamma_{\text{HS}}$) are plotted as a function of temperature, showing an LS‐to‐HS transition while warming up the sample, and in Figure 2h, the HS‐to‐LS transition while cooling. It can be seen that no complete spin‐state switching is accomplished by Fe‐bipyacbipy neither for the thin film nor for the dropcast sample. The maximum HS fractions obtained from Figure 2a–d are 72.4, 71, and 68.4% at 300 K for the three samples, respectively.

The minimum HS fraction of the dropcast sample is $\gamma_{\text{HS}}\approx 34\%$ at 80 K, which is similar to the bulk reference of 25% at 50 K.^[^ ^25^ ^]^ For the 0.7 ML and 1.8 ML samples, the values of *γ*_HS_ at 80 K are significantly higher, with $\gamma_{\text{HS}}\approx 58\%$. The spectra measured at 10 K show that the molecules are susceptible to X‐ray exposure. Therefore, the scanning time of about 110 s for 700–712 eV partly excites the molecules to an HS state due to SOXIESST, which is prominent below 80 K. The higher fraction of HS at 10 K in Figure 2g is due to the exposure of the sample position to X‐rays before starting the heating cycle. To overcome this effect, which is more pronounced at lower temperature, the 1.8 ML sample was also measured during the cooling cycle (Figure 2d,h). The HS fraction for the thin‐film samples at 10 K rises from about 62% (Figure 2f) to about 67% (Figure 2h) due to SOXIESST.

Due to the deposition technique, the dropcast sample builds small islands and not uniform layers; therefore, the interaction with the surface layer is smaller than for the 0.7 ML and 1.8 ML samples. Hence, the dropcast sample has more similar properties to the bulk reference, which also indicates that the SCO properties of the complex are not affected by the presence of the solvent during deposition. Partial or complete loss of switching capabilities in thin films on HOPG was reported earlier, which mainly arises due to the π−CH interaction with the HOPG substrate.^[^ ^5^, ^35^, ^36^, ^37^ ^]^

Due to the higher number of accessible vibrational states and the higher electronic degeneracy of the HS state, the SCO is driven by the entropy difference Δ*S* between the HS and LS states. *γ*_HS_ for the thermal equilibrium of HS and LS can be calculated using a modified van't Hoff equation^[^ ^20^ ^]^

(1) $$\gamma_{\text{HS}}=k_{T}+(c_{T}-k_{T}){\left(\exp\left(\frac{\Delta H}{RT}-\frac{\Delta S}{R}\right)+1\right)}^{-1}$$

where *c*_T_ is the upper saturation and *k*_T_ the minimum *γ*_HS_ fraction, corresponding to the fraction of molecules that cannot be switched. Δ*H* is the enthalpy difference between HS and LS states, and *R* is the gas constant. The residual HS fraction at low temperature is due to some of the molecules being in the HS‐HS or the HS‐LS state. An energetic stabilization of the mixed HS‐LS state has been evidenced and discussed for many dinuclear Fe(II) SCO complexes.^[^ ^27^, ^38^, ^39^, ^40^ ^]^ Therefore, a certain fraction of the molecules may become trapped in the HS‐LS state at low temperatures.

For all three samples, Δ*S* and Δ*H* obtained from fits to the experimental data of Figure 2e–h and represented in **Table** **1** are comparable with the bulk reference values $\Delta S=43(6)\;\;{\text{J K}}^{-1}{\text{mol}}^{-1}$ and $\Delta H=5.4(7)\times 10^{3}\;{\text{J mol}}^{-1}$.^[^ ^25^ ^]^ Using the relation $T_{1/2}=\Delta H/\Delta S$, the $T_{1/2}$ values for the heating and cooling branches have been evaluated (Table 1). The resulting $T_{1/2}=147(13)\;\text{ K}$ for the dropcast sample is close to the value obtained from the XAS measurements of the molecules in bulk form of $T_{1/2}=122\;\text{ K}$ and by PPMS measurement of $T_{1/2}=125\;\text{ K}$, and in good agreement with the UV/Vis result of $T_{1/2}=131\;\text{ K}$.^[^ ^25^ ^]^

**Table 1:** Entropy Δ*S* and enthalpy Δ*H* differences for the measured samples, as well as the upper and lower limit of the HS fraction *c*_T_ and *k*_T_, shown in Figure 2d–f.

	$\Delta S\;[{\text{J K}}^{-1}{\text{ mol}}^{-1}]$	$\Delta H\;[{\text{J mol}}^{-1}]$	*k*_T_	*c*_T_	$T_{1/2}\;[\text{K]}$
Dropcast^[H]^ a)	41(3)	6.1(4) × 10^3^	0.34(1)	0.76(1)	147(13)
1.8 ML^[C]^	35(9)	5.7(12) × 10^3^	0.58(1)	0.70(1)	161(54)
1.8 ML^[H]^	35(14)	6(2) × 10^3^	0.59(1)	0.70(1)	165(87)
0.7 ML^[H]^	39(5)	5.8(6) × 10^3^	0.56(1)	0.73(1)	148(25)

a) [C], cooling cycle; [H], heating cycle.

### 2.2 SOXIESST

**Figure 3:**
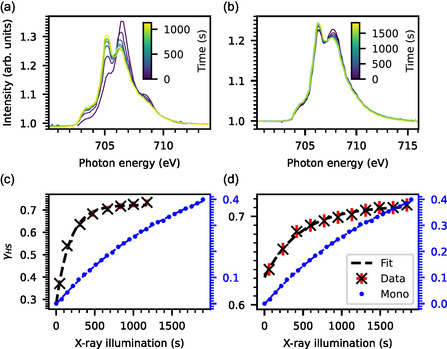
SOXIESST XAS spectra. a) Dropcast sample and b) 1.8 ML. Displayed is the evolution of the XAS spectra undergoing an LS‐to‐HS SCO caused by constant X‐ray illumination at a sample temperature of 10 K. *γ*_HS_ of SOXIESST measurements c) Dropcast sample d) 1.8 ML. Shown is the evaluated *γ*_HS_ from the corresponding XAS spectra. The data is fitted with Equation (2); the parameters are collected in Table 2. The *γ*_HS_ evolution of the 0.7 ML mononuclear complex on HOPG is shown in (c) and (d) as blue dots (scale to the right); the data is taken from Kipgen et al.^[^ ^41^ ^]^ This data has been measured at the VEKMAG endstation.

The temperature‐dependent measurements show that the molecules are susceptible to X‐rays. Therefore, the rate of the SOXIESST effect was measured by constant illumination (700–716 eV) at a sample temperature of 10 K. The illumination time is the accumulated scan time of the Fe *L*_3_ edge, where each scan is assumed to be pre‐exposed to X‐rays for about 60 s. The XAS spectra taken from the dropcast and the 1.8 ML sample are shown in **Figure** **3**a,b. The evaluated *γ*_HS_ are displayed in Figure 3c,d. An exponential function of the form

(2) $$\gamma_{\text{HS}}=c_{S}-\exp(-t_{x}r_{x})\cdot (c_{S}-k_{S})$$

can be fitted, where *t*_*x*_ is the illumination time, *r*_*x*_ the rate constant, *c*_S_ the saturation level for SOXIESST, and *k*_S_ the amount of molecules in the HS state at t=0. The resulting parameters are shown in **Table** **2**. To compare the SOXIESST rates with the mononuclear complex, the data of 0.8 ML thin film of Fe(bpz)‐bipy on HOPG^[^ ^41^ ^]^ is fitted with Equation (2). The obtained value of $r_{x}=5.5(2)\times 10^{-4}{\text{ s}}^{-1}$, measured at a comparable X‐ray flux, confirms the high X‐ray sensitivity of Fe‐bipyacbipy. The data shows that the SOXIESST effect saturates at about 71%. Furthermore, the saturation levels and rates agree with the bulk at c=69(2)% and $r_{x}=2.2(2)\times 10^{-3}{\text{ s}}^{-1}$ for the same X‐ray flux density.^[^ ^25^ ^]^

**Table 2:** SOXIESST rate *r*_*x*_ at an X‐ray photon flux of $5\times 10^{9}\;{\text{ s}}^{-1}{\text{ mm}}^{-2}$ fitted with Equation (2). *c*_S_ is the upper saturation and *k*_S_ the minimum *γ*_HS_ value, data plotted in Figure 3. (Dropcast and 1.8 ML: dinuclear complex).

	*r*_*x*_ [s^−1^]	*c*_S_	*k*_S_
Mononuclear	5.5(2) × 10^−4^	0.61(1)	0
Dropcast	5.6(5) × 10^−3^	0.72(1)	0.28(2)
1.8 ML	2.1(2) × 10^−3^	0.712(2)	0.631(3)

In order to account for the higher SOXIESST rate in the dimer as compared to the monomer, two explanations may be invoked. In an earlier report on a mononuclear complex, the mechanism for SOXIESST has been identified as the generation of secondary electrons from the molecular layer and the substrate after exposure to X‐rays.^[^ ^41^ ^]^ For the mononuclear complex, there is a finite probability that the first electron will cause SOXIESST and further electrons will cause either SOXIESST or reverse SOXIESST, depending upon if an electron hits the LS or the HS complex. In a mixed state of a dinuclear complex, where one Fe is in the HS and the other in the LS state, an electron hitting the LS center would switch to HS–HS as fast as from LS–LS to LS–HS. In coupled Fe(II) SCO systems, electron‐induced SCO may also occur via the transfer of the excitation energy from one Fe(II) center to a neighboring one.^[^ ^42^ ^]^ For a mixed state, if the secondary electron hits the HS center, it is thus conceivable that, due to the coupling of the two iron centers via the bridge, the electron is transferred to the LS center, which switches the complex to the HS‐HS state. This also implies that the reverse SOXIESST rate for the mixed state might be negligible, which would result in higher rates of SOXIESST for the dinuclear complex as compared to the mononuclear complex.

Alternatively, the higher SOXIESST rates for the dinuclear complex as compared to the mononuclear complex could indicate that the absorption cross‐section for secondary electrons is intrinsically higher for the former than for the latter complex. Due to the different film thickness measured in the case of mononuclear and the dinuclear complex, it is difficult to indicate the exact number by which the SOXIESST rates differ. Nevertheless, for both the dropcast sample and the 1.8 ML sample, significantly higher values have been found for *r*_*x*_ than for the monomer. It has been discussed that secondary electrons created within the molecular layer itself have a larger influence on the SOXIESST rate than secondary electrons from the underlying substrate.^[^ ^41^ ^]^ This effect may even be stronger if two neighboring complexes are covalently linked. It should be noted, however, that it also has been found that the energy of the X‐ray irradiation and the HS fraction generated by SOXIESST are not correlated,^[^ ^43^ ^]^ which would render the latter explanation doubtful.

### 2.3 LIESST

**Figure 4:**
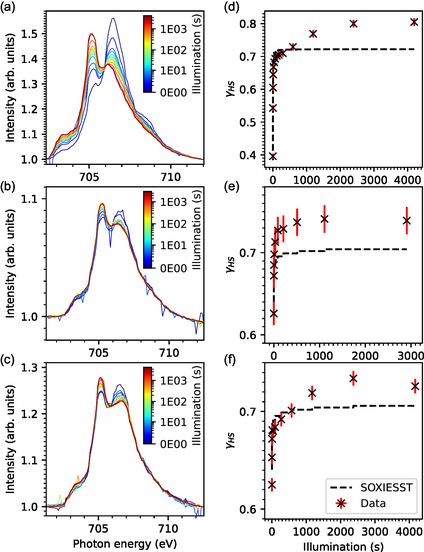
LIESST measurement at 10 K. a,d) Dropcast; b,e) 0.7 ML; and c,f) 1.8 ML. XAS spectra in (a)–(c); *γ*_HS_ as a function of accumulated illumination time in (d)–(f). The black dotted lines in (d)–(f) show the expected SOXIESST effect, calculated with Equation (2) and the values from Table 2. The time axis for the SOXIESST effect is not shown, each scan adds about 100 s X‐ray exposure, while the first scan has half of the exposure time. This data has been measured at the VEKMAG endstation.

To investigate the switching response of the complex to visible‐light exposure, XAS spectra were measured after illuminating the samples with a green LED of wavelength 520 nm to determine the LIESST rate. **Figure** **4**a–f shows the measured XAS spectra and the extracted *γ*_HS_ values. Since the molecules are susceptible to X‐rays, it must be assumed that a significant portion of the switching effect is induced by X‐rays. The black dotted lines in Figure 4d–f show the SOXIESST effect modeled by taking the rate of SOXIESST determined by the single exponential function in Equation (2) and the corresponding parameters in Table 2 (for the 0.7 ML and the 1.8 ML samples, the same values are used). Under this assumption, the data shows for the dropcast, 0.7 ML, and 1.8 ML samples a combined LIESST and SOXIESST effect that is clearly higher than what could be expected by SOXIESST alone. To roughly calculate the LIESST effect in the dropcast sample, *γ*_HS_ can be reduced by the estimated SOXIESST effect with the values from Table 2. To take any offset of slightly different pre‐conditions into account, only the relative change $\Delta \gamma_{\text{HS}}$ is examined. The so‐determined values (Figure S2, Supporting Information) are then fitted with

(3) $$\gamma_{\text{HS}}=c_{L}-\exp(-t_{L}\cdot r_{L})\cdot (c_{L}-k_{L})$$

*t*_L_ is the laser exposure time, *r*_L_ is the rate constant for LIESST, *c*_L_ is the upper saturation, and *k*_L_ the minimum $\Delta \gamma_{\text{HS}}$ value. This gives a rough approximation for $r_{L}=6(2)\times 10^{-4}{\text{ s}}^{-1}$, $k_{L}=0$ and $c_{L}=0.12(1)$. Comparing *r*_L_ to the thin film of the parent molecule with $r_{L}=4.9(2)\times 10^{-2}{\text{ s}}^{-1}$,^[^ ^20^ ^]^ recorded with a laser setup of the same wavelength and similar photon flux, indicates that the LIESST effect could be masked by the SOXIESST effect, so that the calculated rate is underestimating the actual LIESST rate.

### 2.4 *T*(LIESST)

**Figure 5:**
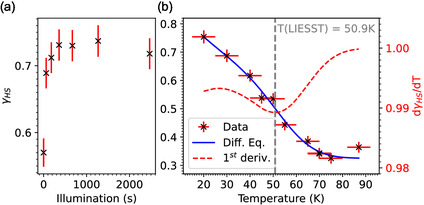
HS fraction of a dropcast sample measured at the PIRX beamline at the SOLARIS synchrotron radiation source. a) The combined LIESST and SOXIESST effect for the sample illuminated by a 520 nm laser LED at 20 K. b) The heating cycle of the sample after illumination. The blue line is the fit of Equation (4), and the red curve is the first derivative. *T*(LIESST) is marked with the dotted gray line.

**Figure** **5**a shows the LIESST measurements of the dropcast sample investigated at the PIRX beamline (SOLARIS) with the green LED of wavelength 520 nm with a photon flux of $6.8\times 10^{10}\;s^{-1}{\text{mm}}^{-2}$. The maximum HS fraction obtained is close to the value obtained after SOXIESST. *T*(LIESST) was determined using the differential Equation (4).^[^ ^44^ ^]^

(4) $$\frac{d\gamma_{HS}}{dT}=-\frac{\gamma_{\text{HS}}}{\theta }\left[k_{\text{HL}}(T\to 0)+k_{\infty }\cdot \exp\left(\frac{-E_{a}}{RT}\right)\right]$$

$E_{a}$ is the activation energy, $k_{\infty }$ is the thermally activated relaxation at high temperatures and at low temperature, assuming monoexponential behavior, the dominant contribution is from the temperature‐independent tunneling rate [$k_{\text{HL}}(T\to 0)$]. The resulting fit parameters are *T*(LIESST) = 50.9 K, $E_{a}=1.8(7)\;{\text{kJ mol}}^{-1}$, $k_{\text{HL}}(T\to 0)=2.8(8)\times 10^{-4}{\text{ s}}^{-1}$, and $k_{\infty }=5(8)\times 10^{-2}{\text{ s}}^{-1}$. Interestingly, the relaxation of the HS state with temperature (Figure 5b) shows a *T*(LIESST) value of 50.9 K, which is close to an earlier report on the bulk complex.^[^ ^25^ ^]^ The results indicate that the complex is sensitive to light, although it is not possible to exactly determine the LIESST rate, as the SOXIESST effect is dominating. The measurements in Figure 5 are done at higher X‐ray photon flux than the measurements shown in Figure 4, which may lead to the transition of the metastable HS state to an LS’ state, where LS’ is a thermally irreversible low‐spin state due to the soft‐X‐ray photochemistry (SOXPC) process.^[^ ^45^ ^]^ This could be seen as a decrease in the HS fraction at high illumination time, which, however, is not above statistical significance. SOXPC is the X‐ray‐induced degradation which will trap the complexes in either an HS or an LS state.

Nevertheless, after switching with X‐rays or by light to the HS state, the complex can relax back to the LS state by temperature. The results confirm that the SCO behavior of the complex is intact. The SOXIESST relaxation temperature (80 K) is only a bit higher than the LIESST relaxation temperature (75 K), such that from these two values it is not straightforward to say whether the SOXIESST effect dominates over the LIESST effect.

### 2.5 Temperature‐ and Light‐Induced Spin‐Crossover

**Figure 6:**
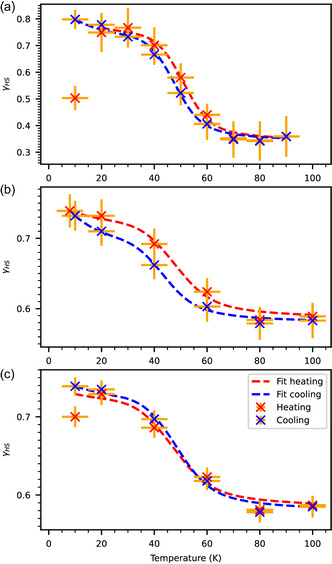
Cooling and heating cycles under constant illumination by green light (520 nm). a) dropcast, b) 0.7 ML, and c) 1.8 ML. Shown is *γ*_HS_ versus the sample temperature. The heating and cooling cycles are fitted with Equation (5) (red and blue dashed lines); the parameters are shown in Table 3. This data has been measured at the VEKMAG endstation.

To determine the kinetics of the HS‐to‐LS relaxation, Fe‐bipyacbipy was measured during heating and cooling cycles between 10 and 120 K under constant laser illumination. The obtained *γ*_HS_ as a function of temperature are shown in **Figure** **6**. The appearance of a hysteresis could be due to a lagging behind of the temperature reading during heating and cooling. As no cooperativity is observed for the investigated samples, the data can be fitted well with Equation (5), limited to a simple Arrhenius behavior, yielding monoexponential isothermal relaxations:^[^ ^44^ ^]^

(5) $$\frac{d\gamma_{\text{HS}}}{dT}=\frac{1}{\theta }\left(k_{\text{exc}}-\gamma_{\text{HS}}\left[k_{\text{exc}}+k_{\text{HL}}(T\to 0)+k_{\infty }\cdot \exp\frac{-E_{a}}{RT}\right]\right)$$

where *θ* is the temperature sweep rate, $k_{\text{exc}}$ is the LIESST excitation rate, $E_{a}$ is the effective energy barrier, $k_{\text{HL}}(T\to 0)$ is the rate of the temperature‐independent relaxation by tunneling, and $k_{\infty }$ is the pre‐exponential factor of the thermally activated relaxation. The data is fitted by solving Equation (5) numerically, using a fixed value of $k_{\text{exc}}=6\times 10^{-4}{\text{ s}}^{-1}$ as calculated by Equation (3) (the light excitation rate (*r*_L_)). The results of the fits are shown in **Table** **3**. The value of *E*_a_ increases with increasing sample thickness, which indicates that the thermal relaxation of the metastable HS state to the LS state gets faster as the thickness of the sample on HOPG decreases.

**Table 3:** Fit parameters for the curves shown in Figure 6 fitted with Equation (5) and fixed $k_{\text{exc}}=6\times 10^{-4}{\text{ s}}^{-1}$.

	*E*_a_ [J mol^−1^]	*k*_HL_ [s^−1^]	*k*_∞_ [s^−1^]	*θ* [K min^−1^]
Dropcast^[H]^ a)	2.5(5) × 10^3^	7.3(71) × 10^−5^	0.7(8)	1.05(2)
Dropcast^[C]^	2.3(3) × 10^3^	1.7(3) × 10^−4^	0.6(4)	−1.05(2)
1.8 ML^[H]^	1.33(3) × 10^3^	1(2) × 10^−4^	0.05(2)	1.57(5)
1.8 ML^[C]^	1.8(6) × 10^3^	1.5(11) × 10^−4^	0.14(17)	−1.36(5)
0.7 ML^[H]^	1.7(5) × 10^3^	2(1) × 10^−4^	0.15(12)	1.45(2)
0.7 ML^[C]^	1.7(3) × 10^3^	3.6(9) × 10^−4^	0.15(12)	−1.11(8)

a) [C], cooling cycle; [H], heating cycle.

## 3 Conclusion

The PLI technique is a new method to deposit large complexes, like Fe‐bipyacbipy, in UHV. The presence of solvent during the deposition does not affect the SCO properties of the dinuclear complex deposited here. The complex in thin films is partially locked in the HS state and possesses a limited switching capability, due to the enhanced interaction with the substrate. Furthermore, it is shown that the characteristics of Fe‐bipyacbipy like entropy and enthalpy differences between HS and LS states remain in the same regime for different thicknesses on HOPG as reported before for a bulk sample.^[^ ^25^ ^]^ A combined effect of SOXIESST and LIESST switches the thin films and a dropcast sample to the HS state at 10 K. The *T*(LIESST) measurement confirms that the HS state relaxes back to the LS state with temperature. The fact that the dinuclear complex Fe‐bipyacbipy is more sensitive to X‐rays than its parent complex on HOPG leads us to assume that there are different mechanisms for the spin‐state switching between the two structures. To determine the LIESST rate more accurately, the X‐ray flux must be significantly lowered, which can be challenging, due to the minimum X‐ray intensity needed to record XAS with sufficient statistics. To the best of our knowledge, this is the first report on thin films of a dinuclear SCO complex deposited as films in the ML regime on surfaces. Utilizing the pulse injection technique can open the door to examining even bigger complexes on different substrates.

## 4 Experimental Section

The PLI system consists of a pulse valve connected to a UHV chamber and is continuously pumped by a turbo‐molecular pump. Above the pulse valve, there is a small reservoir that can hold 0.3 mL of liquid. The pulse valve can open in time intervals in the range from 2 to 100 ms, during which liquid is injected into the chamber. It forms a cone‐shaped shower of droplets and molecular spray.^[^ ^46^ ^]^ This system allows to deposit Fe‐bipyacbipy dissolved in dry tetrahydrofuran (THF) in (sub‐)ML thickness onto HOPG. An inert atmosphere is required to protect the complex from oxidation if it is dissolved for an extended period of time. The molecule is dissolved in 10 mL of dry THF (concentration 2.9 μmol) at a temperature of 75 °C for 45 min under constant stirring.

We also prepared a bulk sample on HOPG by directly dropping the same solvent used for the thin film onto HOPG (dropcast sample). The sample is transferred to the main XAS measurement chamber after having been exposed to atmosphere for about 24 h. The dinuclear complex did not oxidize utilizing this method, since the THF evaporates almost completely within a few seconds. For the thin‐film preparation, we deposit with an accumulated deposition time of 3 s for 0.7 ML and 6 s for 1.8 ML in 20 ms pulses. To ensure a homogeneous sample thickness, three different positions under the pulse valve are used (center, off‐center top, and bottom). The base pressure of the deposition chamber is in the order of $10^{-8}\;\text{ mbar}$, and the peak pressure during injection is in the order of $10^{-2}\;\text{ mbar}$. A driving gas (Argon) of 1 bar is used in the liquid reservoir. The HOPG substrate with dimensions 12×12×2 mm is prepared by removing the top layer with a carbon tape at the cleaving stage of the PLI chamber in about $10^{-7}\;\text{ mbar}$ vacuum. This is done to ensure a clean and flat surface. After deposition, the sample is transferred under UHV conditions to the measurement chamber of the VEKMAG endstation at BESSY II. The AFM measurements were done with a Nanotec Cervantes at room temperature in tapping mode; XAS measurements are carried out with linearly *p*‐polarized light at the PM2‐VEKMAG beamline with a photon flux of $5\times 10^{9}\;s^{-1}{\text{ mm}}^{-2}$ and at the PIRX beamline of the SOLARIS synchrotron radiation source^[^ ^47^, ^48^ ^]^ with a flux of $6.8\times 10^{10}\;{\text{ s}}^{-1}{\text{ mm}}^{-2}$. The energy resolution of the scans is 0.1 eV. The sample is probed using light incidence under the magic angle.^[^ ^49^ ^]^ For the LIESST, a green laser diode with a wavelength of 520 nm and a photon flux density of about $4\times 10^{14}{\text{ s}}^{-1}{\text{ mm}}^{-2}$ at the sample surface is used. The SOXIESST^[^ ^43^ ^]^ measurements are carried out by a continuous scan of the Fe *L*_3_ edge, with constant X‐ray illumination at a sample temperature of 10 K.

LIESST and relaxation of LIESST [*T*(LIESST)] measurements were also performed on the dropcast sample at the PIRX beamline, the concentration of the solution is kept at 2.9 μmol. *T*(LIESST) measurements were taken with temperature sweep rates between 1 and 1.6 Kmin^−1^. To analyze the data, a linear background subtraction and a pre‐edge normalization have been performed. The *L*_3_‐edge Fe^2+^ spectra of the temperature‐dependent measurements are scaled such that the area of all measured spectra matches the area of the spectrum at the lowest temperature. This accounts best for thermal drifts of the manipulator, which leads to probing areas of different densities of the complex that are caused by small nonuniformities due to the deposition method. The HS fraction *γ*_HS_ is calculated by comparing the ratio of the main HS and LS peaks and comparing it to a known ratio, calculated by HS and LS spectra as reported in ref. [22].

All measurement data can be found in ref. [50].

## Conflict of Interest

The authors declare no conflict of interest.

## Supporting information

Supplementary Material

## Data Availability

The data that support the findings of this study are openly available in [Refubium] at [http://doi.org/10.17169/REFUBIUM‐46276], reference number [46276].
