# Supplementary material for: Spin‐Crossover in a Dinuclear Iron(II) Complex on Highly Oriented Pyrolytic Graphite: An X‐Ray Absorption Spectroscopy Study
Source: Chemphyschem. 2025 May 7;26(16):e202401081. doi: 10.1002/cphc.202401081 (PMC12388168; doi:10.1002/cphc.202401081)
Supplement: Supplementary file 1 — Supplementary Material [file CPHC-26-e202401081-s001.pdf]

Supplementary

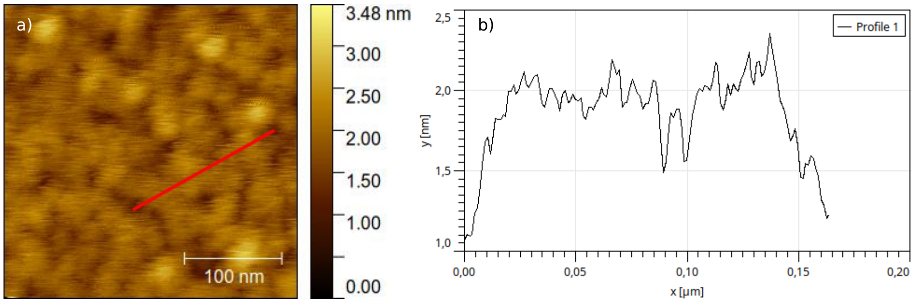

Figure S1: a) Surface topography of 1.8 ML Fe-bipyacbipy on HOPG. The red line marks the position of the height profile displayed in b).

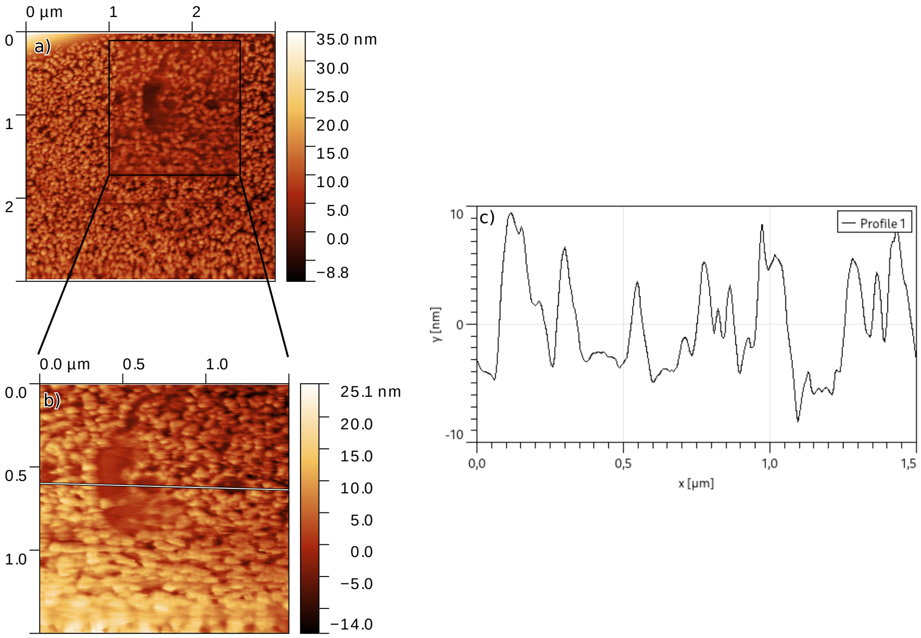

Figure S2: Surface topography of dropcast Fe-bipyacbipy on HOPG. Shown is the formation of islands over a large area a), b) enlarged area marked with a black square in a) and the profile along the white line of b) in c). The middle of b) shows an area with only the HOPG substrate as reference. This strongly suggests that the islands are about 10 nm high.

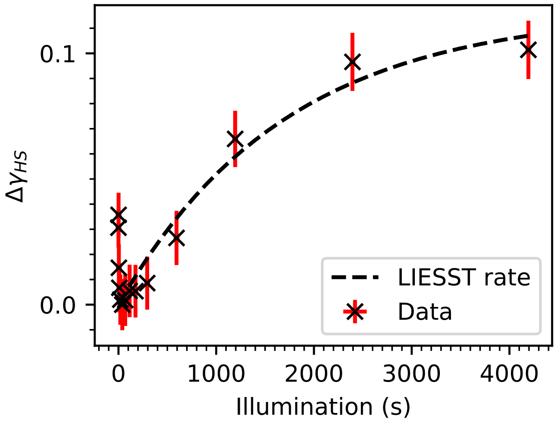

Figure S3: LIESST effect of the dropcast sample reduced by the SOXIESST effect calculated from the X-ray exposure time during the measurement. Shown is the relative change in γ_{HS} after subtraction of SOXIESST as a function of illumination time by a 520 nm laser LED. The values are fitted with Eq. (3). This data has been measured at the VEKMAG endstation.
